# Changes in individual needs for care and quality of life in Assertive Community Treatment patients: an observational study

**DOI:** 10.1186/s12888-014-0306-8

**Published:** 2014-11-18

**Authors:** Hans E Kortrijk, Astrid M Kamperman, Cornelis L Mulder

**Affiliations:** Bavo-Europoort, Prins Constantijnweg 48-54, 3066 Rotterdam, TA The Netherlands; Department of Psychiatry, Epidemiological and Social Psychiatric Research Institute, Erasmus University Medical Centre, ‘s-Gravendijkwal 230, 3015 Rotterdam, CE The Netherlands

## Abstract

**Background:**

It is largely unknown which unmet needs in the Camberwell Assessment of Need Short Appraisal Schedule (CANSAS) need to be resolved in order to improve a patients’ subjective quality of life (QoL). We therefore investigated the pattern of individual unmet needs over time and its relation to QoL over time.

**Methods:**

Using data gathered from 251 patients in a Routine Outcome Monitoring procedure in Assertive Community Treatment (ACT) teams, we used paired samples tests to analyze differences in QoL total scores and the number of unmet needs between baseline and follow-up data. Ordinal regression was used to analyze the relationship between outcome in individual unmet needs and QoL.

**Results:**

As well as small improvements in QoL over time in patients in contact with ACT, we found a small to moderate decrease in unmet needs over time. While a decreasing number of unmet needs was associated with an increase in QoL, outcomes in QoL and individual unmet needs were weakly related (r ≤ .165). Ordinal regression analysis showed that a better outcome in individual unmet needs related to accommodation and day-time activities was weakly related to a better outcome in QoL.

**Conclusions:**

Patients receiving ACT make small improvements in their QoL and ACT may help to solve some of their needs. QoL benefits from reducing needs for care, in particular the need for appropriate housing and meaningful daytime activities.

## Background

Monitoring needs for care and subjective quality of life (QoL) in patients with a severe mental illness (SMI) can be useful in clinical practice [1,2]. It may help prepare and evaluate treatment plans that address interventions that are both individually tailored and negotiated [3]. It also has the potential to increase the effectiveness of mental healthcare treatment [1].

Routine assessment of needs for care provides information on which needs for care are present and on whether they are met or unmet. An *unmet* need, as opposed to a *met* need, indicates that there is a serious problem which was not effectively targeted in treatment. Several studies have found that a patient’s unmet needs for care and changes in these needs are weakly to moderately associated with the patient’s level of QoL, and with changes in this level [4-9]. However, it remains largely unknown which changes in individual unmet needs for care are most strongly associated with changes in QoL.

As needs for care are related to the different stages of a patients’ illness [3], the relation between needs for care and QoL may be very complex. For instance, a patient suffering from acute and severe psychiatric symptoms may have a different pattern of unmet needs – such as needs for help with self-care, safety to self and the treatment of psychotic symptoms – than a patient in symptomatic remission, who may have needs with respect to daytime activities, company and intimate relationships. It is therefore insufficient to study merely the change in the total number of unmet needs, as a different pattern of needs may underlie the total number of needs, thereby concealing changes in individual needs [10]. A more promising method may thus be to study changes in individual met and unmet needs in relation to changes in QoL over time [11].

In addition, because a better understanding of the relationships between individual needs for care and QoL is relevant for the implementation and tailoring of the mental health services, we focused on unmet needs, which, in previous studies, was associated with QoL [6,12]. Although this has been studied relatively extensively over the past decades, a focus on separate components of needs for care is fairly new.

Our objective was to investigate the pattern of individual unmet needs over time and its relation to QoL over time. For this objective, we a) proposed a classification of change and outcome in individual unmet needs and b) calculated criteria for clinically meaningful outcome in QoL by taking account of individual change and the level of QoL.

## Methods

### Setting

Data were collected in the context of a routine outcome monitoring procedure. Assessments were performed by trained independent raters (usually psychologists) and were planned every six to twelve months. These routine outcome monitoring assessments were available for clinicians to use in clinical practice when discussing treatment progress with the patient. Routine outcome monitoring data-collection was approved by the Dutch Committee for the Protection of Personal Data. Data for this study apply to the period from February 2002 to April 2012, and were used anonymously. By Dutch law, studies only using questionnaires, do not need formal evaluation by a Medical Ethical Committee, when data are used anonymously [13]. In addition, in a study such as this, not needing formal evaluation by a Medical Ethical Committee, also no informed consent was required considering the observational nature of the study and because all assessments were collected in the context of a Routine Outcome Monitoring procedure, without any additional burden on the patient.

The study involved patients from seven Assertive Community Treatment (ACT) teams in the city of Rotterdam, the Netherlands. Criteria for treatment by an ACT team were a) age 18 or older, b) having a severe mental illness, usually a psychotic or bipolar disorder (with or without a co-morbid substance use disorder); and c) lack of motivation to be treated at the start of ACT, such that assertive outreach was necessary.

The model fidelity of the ACT teams was assessed using the Dartmouth Assertive Community Treatment Scale (DACTS) [14]. The mean of the total DACTS scores of the ACT teams was 3.5 (range: 2.9 – 3.8), meaning that, on average, ACT had been implemented with moderate success. On the human resources subscale, model fidelity was high (i.e., items that were awarded with scores 4–5). Low scores (i.e., items that were awarded with scores 1–2) were awarded to items pertaining to the nature of services subscale, such as intensity of services, frequency of contact, provision of dual disorder treatment groups, and role of consumers on team (i.e. consumers not involved in providing service) [15].

### Measures

#### Camberwell Assessment of Need Short Appraisal Schedule (CANSAS)

The CANSAS – a modified version of the Camberwell Assessment of Need (CAN) [16] – consists of 22 items [17]. To assess the need for care, it assesses health and social needs across the following domains: accommodation, food, looking after the home, self-care, daytime activities, physical health, psychotic symptoms, information, psychological distress, safety to self, safety to others, alcohol, drugs, company, intimate relationships, sexual expression, childcare, basic education, telephone, transport, money, and benefits. Each item is scored 0 (no problem), 1 (met need) or 2 (unmet need). The reliability of the English version of the CANSAS is acceptable [18,19]. The needs for care were assessed using a Dutch translation of the CANSAS [20,21].

#### Quality of life scale

The Cumulative Needs for Care Monitor (CNCM) quality of life scale was used to measure subjective quality of life (assessed in Dutch) [20,21]. This instrument was based on the Lancashire Quality of Life Profile [22] and was very similar to the Manchester Short Assessment of Quality of Life scale (MANSA) [23], which consists of six items [24]: financial situation, accommodation/living situation, relationship with others, physical health, psychological health, and life as a whole. These are rated on a 7-point scale (1 = “Couldn’t be worse” to 7 = “Couldn’t be better”). This scale has strong correlations with the Lancashire Quality of Life Profile [21].

#### Motivation item

The scale for assessing motivation for treatment was adapted from the Severity of Psychiatric Illness scale [25-27], an observer-rated scale covering the last two weeks. It was scored in five categories (0 = “Highly motivated” to 4 = “Lack of motivation”) in the same way as the Health of the Nation Outcome scales (HoNOS; [28,29]). The psychometric properties of the English and Dutch HoNOS total scores have been found to be acceptable [28,29].

### Analyses

SPSS version 18.0 was used for all analyses. In the routine outcome monitoring data we identified 827 eligible patients, i.e., patients who had had at least two routine outcome monitoring assessments. For analytical purposes we used only complete records of CANSAS (from which no more than 5 items were missing; missing values were then regarded as no need for care) and QoL assessment, and found that 251 patients had completed both the CANSAS and QoL in two consecutive assessments. Descriptive statistics (i.e. means, standard deviations, median, inter-quartile range and percentages) were calculated for outcome variables and patient characteristics. Pearson’s chi-square tests were used for categorical data, and independent samples t-tests for normally distributed data. Paired samples T-tests were used to compare pairwise baseline and follow-up scores for normally distributed data; related-samples Wilcoxon Signed Rank Tests were used for non-normally distributed data. For analytical purposes, the scale for assessing motivation for treatment was dichotomized into two groups (score 0 to 2 and score 3 to 4).

To analyze the associations between changes in QoL over time and changes in the number of unmet needs, we used a regression analysis in which the dependent variable was QoL total score (T1; second available routine outcome monitoring assessment). The determinants were 1) number of unmet needs (T0; first available routine outcome monitoring assessment); 2) QoL total score (T0; first available routine outcome monitoring assessment); 3) changes in number of unmet needs over time, and 4) an interaction (T0 QoL total score * change in number of unmet needs over time).

Then, to study the association between treatment duration and outcome in QoL over time (i.e. to determine whether or not a patient responded to treatment [30]), we determined 1) criteria for clinically meaningful change, and 2) cut-off points between a low and high level of QoL [31]. This combination of both approaches allowed us to look beyond the traditional method of change scores, as it created a hierarchy of outcomes for the QoL scores over time on the basis 1) of the degree of change and 2) of the classification of the total score.

These criteria were calculated using a distribution-based method and an anchor-based method. Distribution-based methods, such as the standard error of measurement (SEM; for formula see Appendix), use statistical characteristics of the data (e.g., standard deviation and measurement precision of the instrument), and provide an estimation of test error which can be used to interpret a patients’ score in a test. The SEM corresponds to a clinically meaningful amount of change in total score (difference between T0 and T1) [30-33].

Anchor-based methods are used to identify cut-off points to differentiate between a low and high level of QoL [33]. Total score cut-off points can be calculated by determining the likelihood that patients who report satisfaction in all QoL domains will exceed that of patients who report dissatisfaction in one or more QoL domain. Patients were considered to have a low QoL if they did not report being satisfied in all the QoL items (i.e., a score below 5 in one or more of the QoL items). The clinical significance (CS) cut-off point between a low and high level of QoL was computed (for formula see Appendix). After these calculations, we combined these criteria (SEM and CS cut-off) to create a model in which meaningful change and a classification of the severity were integrated (according to the Jacobson and Truax approach [34]). By combining the classification (CS-cut-off of 33) and the meaningful change criteria (4-point change), this created 10 possible groups, which were further combined into 4 QoL-change groups for analytic purposes.

The QoL meaningful change and outcome classification was:

1. Very poor: WORSENED from high to low quality of life; WORSENED within low quality of life
2. Fair to poor: STABLE within low to high quality of life; STABLE within low quality of life; STABLE within high to low quality of life
3. Good: IMPROVED within low quality of life
4. Very good: STABLE within high quality of life; IMPROVED within high quality of life; IMPROVED from low to high quality of life

To investigate the change and outcome in individual unmet needs for care (score 2 on individual CANSAS items) in relation to 1) level of QoL and 2) meaningful change in QoL, we created 4 groups for each CANSAS item.

Classification of change and outcome in individual CANSAS items:

1. Very poor: T0 unmet need & T1 unmet need
2. Poor: T0 no unmet need & T1 unmet need
3. Good: T0 unmet need & T1 no unmet need
4. Very Good: T0 no unmet need & T1 no unmet need

To examine the relation between change and outcome in individual CANSAS items (group 1–4) and meaningful change and outcome in QoL (groups 1–4), we used bivariate Spearman correlation coefficients (22 correlations).

After these preliminary analyses, we performed an ordinal regression analysis that included CANSAS items as determinants (22 items; each categorized into 4 groups) and QoL as dependent variable (the 4 groups above). The ordinal regression started with stepwise forward selection in which determinants required a probability value of P < 0.25 for entry into the model. Then, using stepwise backward elimination and a log likelihood test, the determinants were removed at a probability value of P > 0.05 [35]. As two determinants (CANSAS items 10 ‘safety to self’ and 21 ‘money’) violated the proportional odds assumption, they were excluded from the model-fitting procedure.

## Results

### Patient characteristics

Most of the 251 patients who were selected for this study were male (73%). Their mean age was 40 years, and most had been diagnosed with a psychotic disorder (62%). Thirty-four percent were diagnosed with a substance-use-related disorder and 10% with a mood disorder. Twenty-four percent had a missing or deferred diagnosis. Patients had a median number of 5 unmet needs at T0 and a mean QoL total score of 25.9 (SD = 7.8). The duration of follow-up was .9 years (SD = .5).

There were some clinical differences between patient characteristics in both samples (selected (N = 251) and non-selected (N = 576)). More of the patients who were included had been diagnosed with a substance-use-related diagnosis (Pearson’s chi square =14.073, df = 1, p < .05; OR =1.745; 95% CI 1.342 – 2.584). The patients who were included were also more motivated for treatment then those who were excluded (Pearson’s chi square =31.811, df = 1, p < .05; OR =2.787; 95% CI 1.935 – 4.013). There were no other significant differences between the patient samples (sex, age, psychotic disorders, mood disorders, and number of unmet needs at T0).

### Changes in quality of life

Over time, the mean QoL total score increased from 25.9 (SD = 7.8) to 28.6 (SD = 7). A paired samples T-test showed that this was a significant improvement (t = −5.712, df = 250, p < .001). According to Cohen’s rule-of-thumb, this corresponds to a small effect size (d = .36) [36].

We calculated a cut-off score of 33, which indicated that 201 patients had had a low QoL at T0, and 50 patients a high QoL. At T1, 186 patients had a low QoL and 65 a high QoL; 99 (39.4%) had improved in their QoL score (i.e. a positive change of 4 or more points); and 115 (45.8%) had remained stable (a change score within the range of −3 to +3). In 37 patients (14.7%), QoL score deteriorated (i.e. a negative change of 4 or more points).

**Figure 1:**
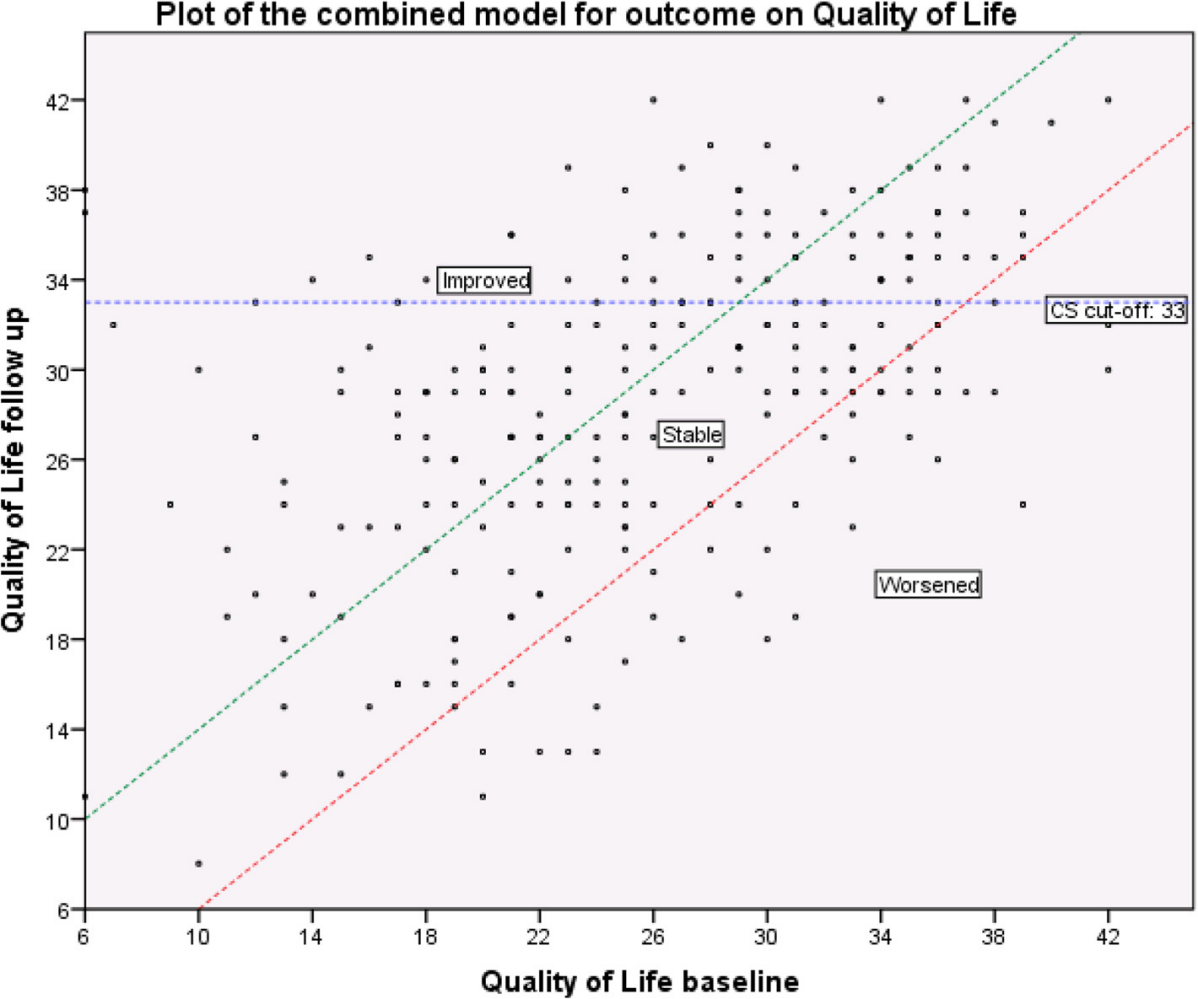
**Outcome on quality of life, a combination of SEM and CS cut-off.** Legend: The horizontal axis represents the baseline Quality of Life (QoL) total score and the vertical axis represents the follow up QoL total score. Each data point represents the combination of the baseline and follow-up QoL total score and illustrates if QoL improves, stabilizes, or worsens over time. The horizontal line represents the clinical significance cut-off between low level of QoL and high level of QoL at follow-up. The diagonal lines represent meaningful change in QoL total score.

Figure 1 presents a scatterplot showing the longitudinal changes in the 251 ACT patients’ QoL total scores. The SEM (meaningful change) and clinical significance (CS) cut-off have both been plotted. Distribution of the QoL change and outcome classification was as follows:

1. Very poor: WORSENED from high to low quality of life; WORSENED within low quality of life (N = 37 (14.7%))
2. Fair to poor: STABLE within low to high quality of life; STABLE within low quality of life; STABLE within high to low quality of life (N = 89 (35.5%))
3. Good: IMPROVED within low quality of life (N = 65 (25.9%))
4. Very good: STABLE within high quality of life; IMPROVED within high quality of life; IMPROVED from low to high quality of life (N = 60 (23.9%))

### Changes in total unmet needs for care

Intercorrelations among outcomes on CANSAS variables turned out to vary substantially, from non significant (165 out of the 231 possible correlations) and small to large. While the size of most correlations (142) lay below .1, eighty-two were in the range of .1 - .3 (small), and only six were in the range of .3 - .5 (moderate). One was > .5 (large).

The five unmet needs reported most at T0 and T1 were daytime activities (T0 74%; T1 57%); company (T0 72%; T1 62%); intimate relationships (T0 52%; T1 45%); psychological distress (T0 39%; T1 31%); and psychotic symptoms (T0 34%; T1 27%).

At T0 the median number of unmet needs was 5 (IQR: 3–7); at T1 it was 4 (IQR: 2–5). To analyze change in the number of unmet needs over time, we used a related-samples Wilcoxon signed-rank test, which showed that the number of unmet needs over time declined significantly (Z = −6.951; p < 0.001). According to Cohen’s rule-of-thumb, this corresponds to a moderate effect size (r = .38).

### Changes in total needs for care and its association with quality of life

Using regression analysis, we studied whether change in the number of unmet needs over time or an interaction between quality of life at baseline and change in the number of unmet needs over time predicted quality of life at follow up (T1). In this regression analysis we corrected for baseline QoL (T0) and the number of unmet needs (T0). The regression analysis showed that change in the number of unmet needs (determinant variable) was significantly associated with QoL at T1, a decrease in the number of unmet needs predicts higher QoL T1 scores. The negative interaction term suggests that the decrease in unmet needs in patients with high T0 QoL scores was less positively associated with QoL at T1. As calculated by *r2* of .423, this set of determinants had a large impact on QoL total score at T1 (Table 1).

**Table 1:** **Linear regression analysis of Quality of Life scores at follow-up**

**Parameter**	**Unstandardized coefficients**	**Standard error**	**Standardized coefficients**	**P**	**95% confidence interval**
*Constant*	19.59	1.802		*<.001*	*16.04 - 23.139*
*N unmet needs baseline*	-.96	.17	-.418	*<.001*	*−1.295 - -.626*
*QoL baseline*	.479	.051	.533	*<.001*	.378 - .58
*Change in N unmet needs* ^*^*^	2.166	.315	1.02	*<.001*	1.546 - 2.786
*QoL baseline Change in N unmet needs*	-.047	.012	-.512	*<.001*	-.071 - -.023

Quality of Life (QoL); ^^^positive change indicates fewer needs at follow-up; R2: .423; F (df = 4, df = 246) = 45.148, p < .001.

### Changes in individual needs for care and quality of life

Table 2 shows the Spearman correlation coefficients between the change and outcome classification on individual CANSAS items (group 1–4; ranging from very poor to very good outcome) and the QoL meaningful change and outcome classification (group 1–4; ranging from very poor to very good outcome). We computed the correlations between 22 individual CANSAS items and QoL. The positive correlations for six items (accommodation, self-care, daytime activities, psychotic symptoms, company and money) with QoL suggested an association between the need for care in question and QoL. The strength of the associations between individual CANSAS items and on QoL ranged from very weak (14 items) to weak (8 items).

**Table 2:** **Correlation between outcome in individual CANSAS items (range 1–4: very poor to very good) and outcome in QoL (range 1–4: very poor to very good)**

1. accommodation	.13^*^
2. food	-.004
3. looking after the home	.034
4. self-care	.147^*^
5. daytime activities	.165^**^
6. physical health	.031
7. psychotic symptoms	.137^*^
8. information	.085
9. psychological distress	.11
10. safety to self	.102
11. safety to others	-.057
12. alcohol	.042
13. drugs	.036
14. company	.148^*^
15. intimate relationships	.072
16. sexual expression	.002
17. childcare	-.087
18. basic education	-.063
19. telephone	.092
20. transport	.055
21. money	.125^*^
22. benefits	.063

^*^Correlation is significant at the 0.05 level (2-tailed). ^**^Correlation is significant at the 0.01 level (2-tailed).

Due to the risk of false positives brought by our computation of 22 correlations, we performed a multivariate ordinal regression analysis; see Table 3. The results show that a contribution to a better QoL classification (meaningful change and outcome) was made by having no unmet needs over time, or by having unmet needs resolved (good outcome) on CANSAS items accommodation (marginally significant) and daytime activities (approaching a level of significance). The odds ratios indicate weak associations between individual unmet needs and QoL. As expected, the pseudo R2 (Nagelkerke) (.075) indicated that there is only a weak association between the changes and outcome in individual CANSAS items and meaningful change and outcome in QoL.

**Table 3:** **Ordinal regression analysis of the relation between outcome in QoL and outcome in individual unmet needs**

**Parameter estimates - QoL groups** ^*^						
		**E (beta)** ^**^	**E (Std. Error)**	**Sig.**	**95% confidence interval**	
					**Lower bound**	**Upper bound**
					**E (beta)**	**E (beta)**
Thresholds	Qol 1: very poor	4.043	2.553	.136	0.644	25.383
	Qol 2: fair to poor	25.504	2.602	.001	3.914	166.174
	Qol 3: good	85.153	2.646	.000	12.649	573.268
	Qol 4: very good		-	-	-	-
CANSAS^*^	1. accommodation	1.278	1.128	.042	1.009	1.618
	4. self-care	1.292	1.211	.182	0.887	1.88
	5. daytime activities	1.216	1.107	.054	0.997	1.483
	7. psychotic symptoms	1.217	1.183	.243	0.875	1.693
	14. Company	1.151	1.101	.144	0.953	1.39

^*^Pseudo R-Square (Nagelkerke) = .075; Goodness-of-Fit Pearson Chi-Square = 288.828. df = 265. p = .151.

CANSAS items 10 and 21 were excluded form the modeling process.

^**^The parameter estimates represent the ratio of the odds for very poor to very good QoL outcome (range 1–4) and for very poor to very good outcome on individual CANSAS items (range 1–4).

A ratio above 1.0 means that better outcome on CANSAS items increases the odds of better QoL over time.

## Discussion

Although our results show that QoL improved over time in patients in contact with ACT teams, the effect was small at group level. But this does not mean that functioning on individual level did not change. At an individual level, while QoL improved in 39% of the ACT patients, it remained stable in 46%, while 15% of patients said that their QoL had deteriorated. This finding was paralleled by a small to moderate decline in the number of unmet needs. Importantly, we found evidence that both were related: positive changes in QoL were associated with positive changes in unmet needs. As we had expected on the basis of the range of the QoL instrument, there was an interaction between QoL at T0 (first available routine outcome monitoring assessment) and a decrease in the number of unmet needs. This means that if patients already have high QoL scores at T0, the relation between change in unmet needs and QoL at T1 (second available routine outcome monitoring assessment) is less favorable.

Our finding that a reduction in unmet needs is weakly associated with improvements in QoL is consistent with that of previous studies examining this relationship [5,7,8,12].

### Change in individual unmet needs and QoL

What we found was a pattern of weak correlations between QoL (meaningful change and outcome) and unmet needs (positive change and good outcomes) for accommodation, self-care, daytime activities, psychotic symptoms, company, and money (all of which are related to areas of self-care, mental health and rehabilitation).

These correlations are quite consistent with our findings in the ordinal regression analysis, which indicated a significant relation between QoL (meaningful change and outcome) and unmet needs (positive change and good outcomes) for accommodation and daytime activities. Self-care, information and company were non-significantly related, which may suggest no association or a nonlinear relationship with QoL or an interaction.

Overall, the relation between individual CANSAS items and QoL was only small. As this is consistent with another study on change in QoL [5], it was to be expected. The pattern we found is partly congruous with the results in a Spanish sample [5], which found a significant association between low QoL and the presence of unmet needs with regard to accommodation, daytime activities, company, intimate relationship, and sexual expression. The partial difference between these results may be explained by differences in study design – note, for example, that neither QoL nor unmet needs were assessed longitudinally.

### Clinical implications

We stress that change and outcome with regard to individual unmet needs were associated only weakly with meaningful change and outcome in QoL. Improvement in observer-rated unmet needs may thus account only partly for improvements in quality of life. This may mean that other factors beside the unmet needs assessed in the CANSAS contribute to a high or low QoL. For example, insight into illness may be associated with low quality of life [37].

Many patients have unmet needs at T1, especially with regard to daytime activities, company, intimate relationships, psychological distress and psychotic symptoms (range 27% - 62%). Many unmet needs thus remain unmet, indicating that, despite ACT, many patients with an SMI still suffer from considerable disability. Together with the many minor intercorrelations between outcomes in individual CANSAS items, this finding may mean that outcomes regarding unmet needs are interrelated, in the sense that an unmet need in one area may have a detrimental effect on needs in other areas [38]. For example, due to homelessness (living on the streets or in shelters), patients may have only a limited ability for self-care.

With regard to the objective of helping patients in contact with ACT achieve a better quality of life, clinicians should, whenever there is an unmet need, help them find a safe place to stay, and should help them regain a healthy and productive lifestyle. Although we found only weak associations, clinicians may be able to improve QoL if they cover patients’ needs for accommodation and daytime activities. However, it is shown by the high number of unmet needs at T1 that this will not be easy.

To ensure that the full range of these problems is treated, we believe it is important to routinely assess subjective quality of life and needs for care.

### Strengths and limitations

This study has two notable strengths. First, the statistical analysis we proposed takes account a) of individual change and outcome in QoL, and b) of change and outcome in individual needs for care. This method allows us to determine which unmet needs are most strongly related to QoL. Secondly, our study comprised a sample of difficult-to-engage patients.

The study has five main limitations. First, as an observational study, its design does not enable us to draw any causal inferences. For instance, many patients have contact with other services as well as their ACT – potential influences for which we are unable to control.

Secondly, as patients at the start of ACT may be at their worst, our results may be influenced by regression to the mean, a tendency for high or low scorers to regress to the mean at the second measurement. We therefore recognize that the presence of potentially uncontrolled elements may have influenced outcome.

Thirdly, we used a subjective QoL measure that led collaborative patients to be selected more than others. Because a higher level of psychosocial problems is associated with less motivation for treatment in severely mentally ill patients [27], this may have biased our results (i.e. towards an overestimation of treatment success). However, more of the patients who were included had a substance use disorder, and thus a higher risk of poor treatment outcome [39]. These two factors may have had a counteracting effect on the estimates of treatment outcome. It is also possible that the relationships between QoL and unmet needs differed between patient groups with a dual diagnosis and those without.

In an effort to deal with the missing QoL records, missing data for a subject were replaced by the mean value of the other QoL items from that subject. This method meant that the amount of missingness remained substantial (286 patients out of 827). After imputation, there was therefore no noticeable change in the proportion of patients with a high or low QoL, or in the degree of change (results not shown).

Fourthly, because we calculated the criteria for meaningful outcome (SEM or cut-off scores) on the basis of patients in contact with ACT teams, our classification of outcome on QoL may seem arbitrary. Similarly, as we have found no reference data on meaningful outcome criteria for the QoL items we used, the hierarchy in which we classified QoL outcomes was also arbitrary. Although our results on change and outcome in the CANSAS and QoL are congruous with those of other studies [5-9], we cannot conclude that this validates our proposed methodology. A future validation research with alternative outcome measures may therefore show how our proposed method (classification of QoL) will generalize to an independent data-set.

The fifth limitation is that some of the needs for care items (such as accommodation, company, money, physical health and psychological distress) are also included in the overall QoL measure. Obviously, there is some overlap between the two measures, and correlations are likely between the QoL measure and needs-for-care items, though it is clear from our results on the relationship between needs for care and QoL that not all needs for care are important for quality of life, and certainly not equally important.

## Conclusions

In conclusion, patients receiving ACT make small improvements in their QoL and ACT may help to solve some of their needs. Furthermore, a reduction in unmet needs is weakly associated with improvements in QoL.

More specifically, QoL benefits from reducing needs for care, in particular the need for appropriate housing and meaningful daytime activities.

## Appendix

The SEM was calculated as SD_0_ × √ (1 ‐ α), where SD_0_ is the standard deviation of the T0 observations and α is Cronbach’s coefficient Alpha.

The clinical significance (CS) cut-off point between a low and high level of QoL was computed as follows: ((mean_low_ × SD_high_) + (mean_high_ × SD_low_))/(SD_low_ + SD_high_).
